# *XRCC1* Gene Polymorphism Increases the Risk of Hepatocellular Carcinoma in Egyptian Population

**DOI:** 10.31557/APJCP.2020.21.4.1031

**Published:** 2020-04

**Authors:** Mary Naguib, Mohamed M Helwa, Mohammed M Soliman, Mohamed Abdel-Samiee, Ashraf M Eljaky, Osama Hammam, Hassan Zaghla, Eman Abdelsameea

**Affiliations:** 1 *Department of Clinical Pathology, *; 3 *Hepatology and Gastroenterology, National Liver Institute, *; 2 *Clinical Pathology Department, Faculty of Medicine, Menoufia University, Egypt. *

**Keywords:** XRCC1, SNP, Hepatocellular carcinoma, RFLP, DNA repair

## Abstract

**Section Title:**

Several major risk factors for hepatocellular carcinoma (HCC) have been identified, including chronic infection of hepatitis B virus (HBV) and hepatitis C virus (HCV). Nevertheless, only a fraction of infected patients develops HCC during their lifetime suggesting that genetic factors might modulate HCC development. X-ray repair cross complementing group1 (*XRCC1*) participates in the repair pathways of DNA.

**Aim:**

to investigate the association between *XRCC1* gene polymorphism and HCC in Egyptian chronic hepatitis C patients.

**Methods:**

This study was assessed on 40 patients with HCC secondary to chronic HCV infection who were compared to 20 cirrhotic HCV patients and 40- age and gender- matched healthy control group. After collection of relevant clinical data and basic laboratory tests, *c.1517G>C SNP* of *XRCC1* gene polymorphism was performed by (PCR-RFLP) technique.

**Results:**

A statistically higher frequency of *XRCC1* (CC, GC) genotypes and increased (C) allele frequency in patients with HCC was found in comparison to cirrhotic HCV patients as well as control group. In addition, patients with the *XRCC1* (CC, GC) genotypes had significantly higher number and larger size of tumor foci and significantly higher Child Pugh grades. Multivariate analysis showed that the presence of *c.1517G>C SNP* of *XRCC1* gene is an independent risk for the development of HCC in chronic HCV patients with 3.7 fold increased risk of HCC development.

**In conclusion:**

*XRCC1 *gene polymorphism could be associated with increased risk of HCC development in chronic HCV Egyptian patients.

## Introduction

Hepatocellular carcinoma (HCC) is a worldwide health problem. In Egypt, it represents the second most common cancer in men and the sixth most common cancer in women (Omar et al., 2013). Hepatocellular carcinoma is the third cause of mortality due to cancer (El-Garawani et al., 2020). Several risk factors have been recognized, including chronic infection with hepatitis B virus (HBV) and hepatitis C virus (HCV) (Hasan et al., 2014). Chronic liver inflammation is associated with repair and tissue remodelling processes, which may lead to chromosomal damage and subsequent progression to cirrhosis and initiation of hepatocarcinogenesis (Gao et al., 2012). 

DNA is under constant threat from endogenous and exogenous DNA damaging agents. Highly conserved DNA repair systems are settled to process DNA damage and maintain genomic integrity (Abbotts et al., 2014). Among these, the X-ray repair cross-complementing group1 (*XRCC1*) which is responsible for repair of oxidative DNA damage and single-strand breaks (London, 2015). *XRCC1* acts as a scaffolding protein that interacts with multiple repair enzymes allowing them to carry out their enzymatic steps in repairing DNA (Xu et al., 2015). *XRCC1* gene is located on chromosome 19q13.2-13.3. It spans a genetic distance of 33 kb comprising 17 exons. It encodes a 70-kDa protein which consists of 633 amino acids (Hanssen-Bauer et al., 2012).

Several studies indicated an association between *XRCC1* genetic polymorphisms and a variety of cancers such as gastric (Chen et al., 2016), lung (Cătană et al., 2015), thyroid (Wang et al., 2015) and breast cancer (Bu et al., 2014). This study aimed to investigate *c.1517G>C SNP *of *XRCC1* gene as HCC risk factor in Egyptian population.

## Materials and Methods

*Subjects and Methods*

The present case control study was conducted in the National Liver Institute, Menoufia University in the period from June 2016 to June 2017. A total of 100 subjects; 40 patients with HCC secondary to chronic HCV infection, 20 post hepatitis C cirrhotic patients - with no radiological evidence of HCC- and 40- age and gender- matched healthy control group were enrolled in the study.

The diagnosis of HCC was based on history taking, clinical examination, radiological examination including abdominal ultrasound and tri-phasic computed tomography (CT) of abdomen and laboratory investigations including hepatitis C and B markers and alpha-fetoprotein (AFP) level.

Patients with causes of liver cirrhosis and HCC other than chronic HCV infection were excluded like patients presented with chronic HBV infection, metabolic liver diseases, autoimmune liver diseases, fatty liver disease and alcoholic liver diseases.

The study protocol was approved by the local ethics committee of the Menoufia University. Informed consents were taken from both the patients and control group subjects before the beginning of the study.

*Routine laboratory investigations*

After collection of relevant clinical data, basic laboratory tests were performed including complete blood counts (Sysmex XT-1800i Automated Hematology Analyzer, Sysmex Corporation, Kobe 651-0073, Japan), liver function tests (cobas- 6000 auto analyser, Roche diagnostics- GmbH, D-68305 Mannheim, Germany), prothrombin concentration and international normalized ratio (INR) (BFT II Analyzer, Dade Behring Marburg GmbH, D-35041 Marburg, Germany), hepatitis serology (HBsAg and HCV Ab) and serum α-fetoprotein level (cobas e411 immunoassay analyser, Roche diagnostics- GmbH, D-68305 Mannheim, Germany). 

*DNA extraction and genotyping*

Venous blood sample was drawn from each subject and genomic DNA was extracted using Zymo Quick-gDNA™ MiniPrep DNA Purification Kit (Zymo Research, CA, USA).

*XRCC1 c.1517G>C* polymorphism was detected using polymerase chain reaction-restriction fragment length polymorphism (PCR-RFLP) method as previously described (Bi et al., 2013). The 247 base pair (bp) fragment was amplified using the amplification mix in a total volume of 25μl which consisted of 1 μL of each of primers; forward primer: 5’-CAAGTCCCAGCTGAGAACTGAG-3’ and reverse primer: 5’- GCTGCTCTGCATGCTCACTC -3’, 12.5 μl of MyTaq™ Red Mix master mix (2X) (Bioline, MA, USA), 5.5 μl of nuclease-free water and 5 μl of extracted genomic DNA.

The PCR amplification was performed on pre-programmed thermal cycler (Perkin Elmer Gene Amp PCR System 2400 Thermal Cycler version 2.11, USA) under the following conditions: an initial denaturation step at 94°C for 5 min, followed by 35 cycles, 94°C for 35 seconds, annealing at 59°C for 35 seconds and 72°C for 35 seconds. Then final extension at 72°C for 5 min was carried out.

As a negative control, PCR mix without DNA sample was used to ensure contamination free PCR product. Confirmation of successful PCR amplification was done using 2% agarose gel electrophoresis. Then 10 μl of amplified DNA were digested at 37°C in a heat block for 5-15 min in a reaction mixture containing 1 μl HaeIII enzyme (New England Biolabs, MA, USA). 

**Figure 1:**
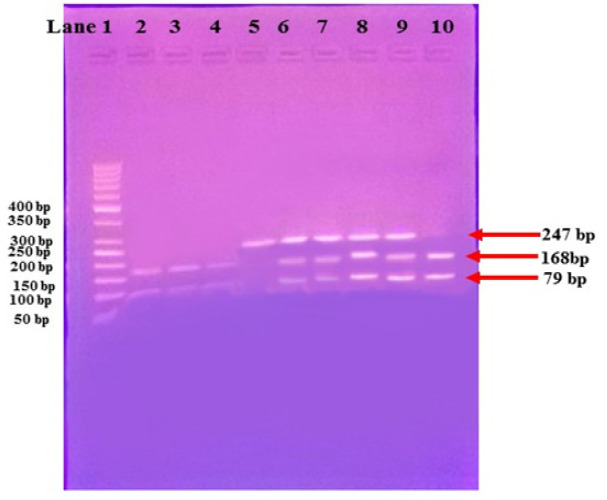
A Representative Agarose Gel Picture Showing PCR-RFLP Analysis of XRCC1 (c.1517G>C) Polymorphism of Studied Subjects after Digestion by HaeIII Restriction Enzyme. Lane 1 50-bp DNA ladder, lane 6, 7, 8 and 9 G/C heterozygous (247, 168 and 79 bp bands), lane 2, 3, 4 and 10 C/C homozygous (168 and 79 bp bands) and lane 5 G/G homozygous (247 bp)

After digestion, fragments were separated on 2% agarose gel. HaeIII digests amplified DNA at the C allele, and yields two fragments; 168 bp and 79 bp. Accordingly, samples yielding 168 and 79 bp fragments were recorded as homozygous CC genotype, those yielding a single 247 bp fragment were recorded as homozygous GG genotype, while those yielding 247, 168 and 79 bp fragments were recorded as heterozygous GC genotype (Figure 1).

*Statistical analysis*

Results were collected, tabulated and statistically analysed by statistical package SPSS version 20 (Armonk, NK; IBM corporation). Data was expressed into two phases: Descriptive (number, percentage, mean and standard deviation) and analytical study (Chi-square test, Mann Whitney test, Kruskal–Wallis test, ANOVA test followed by Post Hoc Test (Dunn’s multiple comparisons test), and Fisher’s Exact test, Odds ratio (OR) and confidence interval (CI) test) were used. p value > 0.05 was considered statistically non-significant and p value < 0.05 was considered statistically significant. 

**Table 1:** Demographic Characteristics of the Studied Groups

	Group I (cirrhosis) (n = 20)		Group II (HCC) (n = 40)		Group III (Control) (n = 40)		Test of significance	P
	No.	%	No.	%	No.	%		
Gender								
Male	16	80.0	36	90.0	28	70.0	c2= 5.00	0.082
Female	4	20.0	4	10.0	12	30.0		
Age (years)								
Range	41.0 - 62.0		42.0 – 63.0		41.0 – 59.0		F= 2.58	0.081
SD ± Mean	51.25 ± 5.32		52.75 ± 4.79		50.48 ± 3.76			
Median	50.50		52.50		51.00			

χ^2^; p, χ^2^ and p values for Chi square test for comparing between the three studied groups; F; p, F and p values for ANOVA test

**Figure 2:**
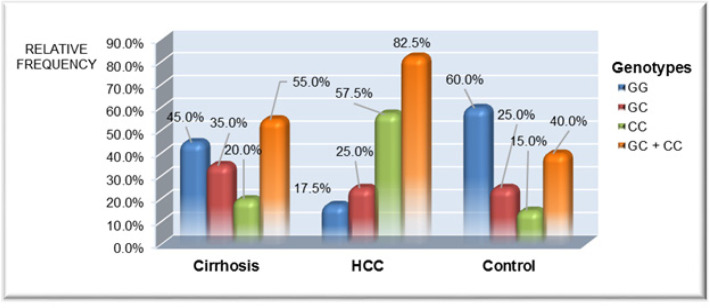
Comparison between the Different Studied Groups as Regard to XRCC1 (c.1517G>C) Genotype Distribution

**Figure 3:**
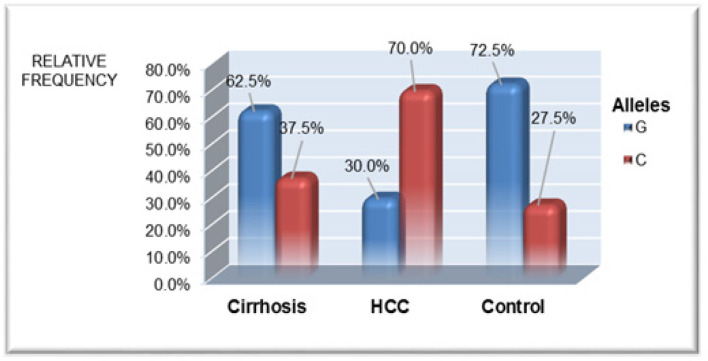
Comparison between the Different Studied Groups as Regard to Allele XRCC1 (c.1517G>C) Frequency Distribution

**Table 2:** Statistical Analysis of the Laboratory Results among the Studied Groups

Laboratory parameters	Studied groups			Test of significance	P value	Significance
	Group I (cirrhosis)	Group II (HCC)	Group III (Control)			
	(n=20)	(n=40)	(n=40)			
Platelets (×10³/µl)						p_1_=0.517
Range	88.0 – 200.0	31.0 – 231.0	152.0 – 340.0	H=50.344*	<0.001*	p_2_<0.001*
Median	126.5	107.5	210.5			p_3_<0.001*
INR						p_1_=0.889
Range	1.12 – 2.54	1.07 – 4.50	1.0 – 1.11	H=69.373*	<0.001*	p_2_<0.001*
Median	1.55	1.58	1.01			p_3_<0.001*
AST (IU/L):						p_1_=0.004*
Range	11.0 – 120.0	35.0 – 2129.0	10.0 – 25.0	H=70.068*	<0.001*	p_2_<0.001*
Median	61.0	109.5	15.0			p_3_<0.001*
ALT (IU/L):						p_1_=0.930
Range	23.0 – 170.0	11.0 – 748.0	10.0 – 25.0	H=57.412*	<0.001*	p_2_<0.001*
Median	45.50	54.50	16.0			p_3_<0.001*
ALP (IU/L):						p_1_=0.128
Range	45.0 – 364.0	92.0 – 728.0	46.0 – 89.0	H=62.466*	<0.001*	p_2_<0.001*
Median	102.8	151.0	64.50			p_3_<0.001*
Albumin (g/dl):						p_1_=0.010*
Range	2.10 – 3.60	1.80 – 3.90	3.70 – 5.0	F=191.817	<0.001*	p_2_<0.001*
Median	2.60	2.20	4.35			p_3_<0.001*
Total bilirubin (mg/dl):						p_1_=0.409
Range	0.40 – 23.20	0.75 – 15.70	0.20 – 0.80	H=62.837*	<0.001*	p_2_<0.001*
Median	3.83	3.44	0.53			p_3_<0.001*
Direct bilirubin (mg/dl):						p_1_=0.614
Range	0.10 – 12.60	0.22 – 12.07	0.07 – 0.20	H=65.883	<0.001*	p_2_<0.001*
Median	1.40	2.08	0.11			p_3_<0.001*
AFP (ng/mL):				H=63.660	<0.001*	p_1_=0.002**
Range	1.63 – 4.0	1.32 – 5882.0	1.0 – 3.0			p_2_=0.001*
Median	2.85	52.0	1.60			p_3_<0.001*

INR, International Normalized Ratio; AST, Aspartate aminotransferase; ALT, Alanine aminotransferase; ALP, Alkaline phosphatase; AFP, alfa fetoprotein; F,p, F and p values for ANOVA test; Significance among groups was done using Post Hoc Test (LSD); H, p, H and p values for Kruskal Wallis test, Significance among groups was done using Post Hoc Test (Dunn's multiple comparisons test); *, Statistically significant at p ≤ 0.05; p_1_, p value for comparing between group I and group II; p_2_, p value for comparing between group I and group III; p_3_, p value for comparing between group II and group III

**Table 3:** Distribution of XRCC1 (c.1517G>C) Genotype and Allele Frequencies among the Studied Groups

	Group I (cirrhosis)		Group II (HCC)		Group III (Control)		χ^2^	p	Significance between groups
	(n = 20)		(n = 40)		(n = 40)				
	No.	%	No.	%	No.	%			
Genotypes									
GG	9	45.0	7	17.5	24	60.0	22.01*	<0.001*	P_1_=0.015*
GC	7	35.0	10	25.0	10	25.0			P_2_=0.493 a
CC	4	20.0	23	57.5	6	15.0			P_3_<0.001*
GG	9	45.0	7	17.5	24	60.0	15.31*	<0.001*	P_1_=0.023* P_2_=0.271 P_3_<0.001*
GC + CC	11	55.0	33	82.5	16	40.0			
Alleles									P_1_=0.001*
G	25	62.5	24	30.0	58	72.5	30.67*	<0.001*	P_2_=0.263
C	15	37.5	56	70.0	22	27.5			P_3_<0.001*

χ^2^, p, χ^2^ and p values for Chi square test for comparing between the three groups; Significance among groups was done using Fisher Exact test; *, Statistically significant at p ≤ 0.05; p_1_, p value for comparing between group I and group II; p_2_, p value for comparing between group I and group III; p_3_, p value for comparing between group II and group III

**Table 4:** Univariate and Multivariate Logistic Regression Analysis for HCC Cases

	Univariate Analysis			
	P value	OR	95% CI	
			Lower	Upper
Age (years)	0.272	1.065	0.952	1.192
Gender (male)	0.291	0.444	0.099	2.004
Viral load	0.096	1.001	1.000	1.002
XRCC1 genotypes (GC+CC)	0.028*	3.857	1.161	12.813
Child Pugh Classification	0.084	3.051	0.862	10.799
(B +C)				
Viral load	0.127	1.001	1.000	1.002
XRCC1 Genotypes (GC+CC)	0.042*	3.742	1.051	13.322
Child Pugh Classification	0.198	2.438	0.627	9.475
(B+C)				

OR, Odds ratio; CI, Confidence interval

**Table 5:** Comparison of XRCC1 Genotypes as Regard to HCC Characteristics

	Results				Test of sig.	P
	GG (n = 7)		GC+CC (n = 33)			
	No.	%	No.	%		
Number of foci						
Single	5	71.4	5	15.2	χ^2^=9.755*	FEp=0.006*
Multiple	2	28.6	28	84.8		
Size (cm)						
Max. – Min.	1.70 – 5.50		2.0 – 11.0		U=33.50*	0.003*
SD ± Mean	2.63 ±1.32		5.30 ± 2.39			
Median	2.50		5.0			
Child Pugh classification						
A	2	28.6	4	12.1	χ^2^=6.08*	^FE^p= 0.035*, a
B	3	42.9	4	12.1		
C	2	28.6	25	75.8		

χ^2^, p, χ^2^ and p values for Chi square test; ^FE^p, p value for Fisher Exact for Chi square test; U, p, U and p values for Mann Whitney test; *, Statistically significant at p ≤ 0.05

## Results

*Demographic and laboratory data of the studied groups*

There was no significant difference among the three studied groups in terms of age and gender distribution (Table 1). However, as shown in Table 2, statistically significant difference between HCC group and control group was detected regarding platelet count, liver tests and AFP. On the other hand, comparing HCC group to cirrhotic group showed significantly higher aspartate aminotransferase (AST) and serum AFP levels and significantly lower albumin levels with no statistical difference regarding platelet count, international normalized ratio (INR), alanine aminotransferase (ALT), total bilirubin, direct bilirubin and alkaline phosphatase.

*XRCC1 genotype distribution and allele frequency among studied groups*

Studying the frequency of different genotypes and alleles of *XRCC1 (c.1517G> C)* polymorphism among different studied groups are shown in Figures 2, 3 and Table 3. Control group showed significantly higher percentage of GG genotype versus HCC group (60% vs. 17.5%, p value < 0.001) with significantly lower percent of C allele (27.5%) versus HCC group (70%, p< 0.001). 

HCC patients had significantly higher incidence of CC and GC genotypes (82.5%) when compared to healthy controls (40%, p< 0.001) and cirrhotic patients (55%, p =0.023) with increased C allele frequency in patients with HCC in comparison to healthy controls as well as cirrhotic patients group (p <0.001 and p=0.001 respectively). 

*XRCC1 gene polymorphism and the risk of hepatocellular carcinoma*

Univariate analysis revealed that the CC, GC genotypes were associated with 3.857 increased risk of HCC compared to GG genotype. The multivariate analysis showed that the presence of *XRCC1* (c.1517G>C) polymorphism is an independent risk for the development of HCC in chronic HCV patients with 3.742 fold increased risk of HCC development (Table 4).

In addition, patients with CC, GC genotypes had significantly higher number of tumor foci (p= 0.006), and larger size of tumor foci (p= 0.003) and advanced Child Pugh grade (p= 0.035) (Table 5). 

In an attempt to study CC homozygous genotype as an independent factor affecting foci lesions in HCC group, we studied characteristics of focal lesions in relation to *c.1517G>C* CC homozygous genotype vs. GC and GG. However, there was no statistical significance between 2 groups regarding number of foci or focal size lesion (p=1.000, 0.805 respectively). In addition, there was no statistical difference regarding Child Pugh classification (p=0.497). 

## Discussion

As a complex and multi-factorial process, both genetic and environmental factors affect liver pathogenesis contributing to carcinogenesis (Parsa, 2012). Identifying those factors could guide understanding various pathways involved in hepatic carcinogenesis, this may improve screening policies for high risk patients. 

DNA repair mechanisms interact to conserve genome integrity and avoid carcinogenesis. Base excision repair (BER) constitutes the primary defense against lesions generated by ionizing radiation and strong alkylating agents, in addition to other DNA-damaging agents as viruses.* XRCC1* gene has been found to play a pivotal role in the base excision repair (BER) pathway. Mutations of *XRCC1* may increase the risk of cancer through impairing its interaction with other enzymatic proteins with consequent impairment of DNA repair activity (Basso et al., 2007; Tudek, 2007).

Previous studies showed significant association between HCC and different SNPs in *XRCC1* gene. Xia et al., (2014) noted that the genotypes and alleles distribution of *XRCC1* variants c.910A>G and c.1686C>G were statistically associated with the risk of HCC. Liu et al., (2014) reported that c.1804C>A genetic polymorphism of *XRCC1* may influence the risk of HCC (Liu et al., 2014). Kiran et al., (2009) found that Arg194Trp and Arg280His genotypes showed an increased risk of HCC which was further enhanced when Arg280His genotype was found in association with Arg194Trp and Arg399Gln. Also, Qi et al., in (2014) and Bazgir et al in (2018) noted that *XRCC1 Arg399Gln* polymorphism was associated with an increased risk of HCC. However, Liu et al., (2011) in their meta- analysis found no association between *Arg399Gln *polymorphism of *XRCC1* and the risk of HCC. Thus these results remain to be elucidated. 

Previously c.1517G>C genetic variant of the* XRCC1* gene also was reported to be significantly associated with pancreatic cancer in a study conducted by Zhao et al., (2014) They noted that The CC genotype was significantly associated with an increased risk of pancreatic cancer. They reported that C allele may contribute to development of pancreatic cancer. 

Our study aimed to investigate the association between *XRCC1 (c.1517G>C)* polymorphism and the risk of HCC in Egyptian patients who are chronically infected with HCV. This genetic variant represents a non-synonymous G to C mutation in exon 14 of the *XRCC1* gene, resulting in glycine (Gly) to alanine (Ala) amino acid replace-ment (p.Gly506Ala) (Zhao et al., 2014). 

We found statistically higher frequency of XRCC1 (CC, GC) genotypes in patients with HCC (82.5%) in comparison to cirrhotic HCV patients (55%) as well as control group (40%) with higher percentage of C allele (70%) in HCC group. The multivariate analysis revealed that the presence of* c.1517G>C SNP* of *XRCC1* gene was an independent risk factor for the development of HCC in chronic HCV patients with 3.7 fold increased risk of HCC development. Furthermore, patients with CC, GC genotypes had significantly higher number and larger size of tumour foci and advanced Child Pugh grades.

This was in agreement with Bi et al., (2013) who studied c.1517G>C and c.1254C>T polymorphisms in XRCC1 gene among HCC Chinese Han population. They found that there was statistically significant association between XRCC1 (CC, GC) genotypes and the risk of HCC. As in HCC group CC, GC and GG genotypes represented 15.21%, 47% and 37.79% respectively, with CC/GC genotypes versus GG genotype OR 1.63 increased risk of HCC; p < 0.001. They reported that the C-allele of c.1517G>C genetic variants may influence the susceptibility to HCC (p< 0.001). They also noted significant association between c.1254C>T polymorphism and HCC risk.

However, there was no statistical significance between c.1517G>C CC homozygous genotype vs. GC and GG in HCC patients group regarding number of foci, focal size lesion or Child Pugh classification.

In conclusion, *XRCC1 (c.1517G>C)* polymorphism could be associated with increased risk of HCV- related HCC development in Egyptian population but the definite association between them needs to be validated in other large multicentre cohort studies.

## Conflict of Interest

Author Mary Naguib declares that she has no conflict of interest. Author Mohamed M Helwa declares that he has no conflict of interest. Author Mohammed M Soliman declares that he has no conflict of interest. Author Mohamed Abdel-Samiee declares that he has no conflict of interest. Ashraf M Eljaky declares that he has no conflict of interest. Author Osama Hammam declares that he has no conflict of interest. Author Hassan Zaghla declares that he has no conflict of interest and Author Eman Abdelsameea declares that she has no conflict of interest.

## Ethical approval

All procedures performed in our study were in accordance with the ethical standards of the institutional and/or national research committee and with the 1964 Helsinki declaration and its later amendments or comparable ethical standards.

## Informed consent

An informed consent was obtained from all individual participants included in the study.

## Ethical approval

All procedures performed in our study were in accordance with the ethical standards of the institutional and/or national research committee and with the 1964 Helsinki declaration and its later amendments or comparable ethical standards.

## Informed consent

An informed consent was obtained from all individual participants included in the study. 
